# Comparison of MynxGrip vascular closure device and manual compression for closure after femoral access angiography: a randomized controlled trial: the closure devices used in every day practice study, CLOSE-UP III trial

**DOI:** 10.1186/s12872-022-02512-0

**Published:** 2022-02-23

**Authors:** Lars Jakobsen, Niels R. Holm, Michael Maeng, Troels Thim, Steen D. Kristensen, Lone H. Mogensen, Evald H. Christiansen

**Affiliations:** Department of Cardiology, Aarhus University Hospital, Palle Juul-Jensens Boulevard 99, 8200 Aarhus N., Denmark

**Keywords:** Femoral, Vascular access closure device, Access site, Bleeding

## Abstract

**Background:**

Complications related to femoral artery access for coronary angiography (CAG) is a safety concern. Vascular closure devices (VCDs) have been developed to reduce the rate of complications after femoral artery access. We compared the safety and efficacy of the MynxGrip VCD versus manual compression (MC) after femoral access CAG in a randomized controlled trial.

**Methods:**

The study was a randomized, single center, non-blinded, two-arm non-inferiority trial. The study was stopped prematurely because of low inclusion rate.

**Results:**

We randomized 869 patients to closure with the MynxGrip VCD or MC and 865 entered analyses. The incidence of the primary endpoint of major adverse vascular events (MAVE) after 30 days was 1.2% in the MynxGrip group and 0% in the MC group (p = 0.06). The median time to hemostasis was 4 [3:5] minutes and 10 [7:11] minutes in the MynxGrip group and MC group, respectively (p < 0.0001). The corresponding median times to mobilization was 73 [65:87] minutes and 76 [70:88] minutes (p = 0.01).

**Conclusions:**

MAVE was rare after closure of femoral arterial access by both the MynxGrip VCD and MC. We found a numerical difference in favour of MC but this did not reach statistical significance. Time to hemostasis was shorter in the MynxGrip group when compared to the MC group.

***Trial registration*:**

The study was approved by the local medical ethics committee and registered at clinicaltrials.org (ClinicalTrials identifier: NCT02237430 11/09/2014).

## Introduction

Coronary angiography (CAG) is the standard procedure for invasive evaluation of coronary artery disease. Although the use of radial access for CAG is increasing, femoral artery access is still extensively used as access site for CAG. The principal safety concerns when performing femoral CAG are complications related to femoral artery access, such as bleeding, groin hematoma, pseudoaneurysm, and stenosis or closure of the femoral artery [1]. In 2014, Ortiz et al. [2] reported a complication rate of 3.5% of which most were minor complications (74.4%). However, 9.7% required blood transfusion, 5.4% required thrombin injection, and 10.5% required surgery. These complications lead to longer hospital stay or discharge to nursing facilities resulting in higher hospital costs. In addition, compared to patients without access site complications patients with severe access site complications had higher 30-day mortality (6.1% vs 1.4%; P < 0.001), and those with moderate complications requiring transfusion had 1-year mortality rate of 12.1% (vs 5.7% without complication; P < 0.001). Manual compression (MC) has remained the “gold standard” for achieving hemostasis; however, this can be time-consuming and personnel intensive (10–20 min or more of MC), requires prolonged bed rest upon completion, and can be uncomfortable for both the patient and the provider. Consequently, closure of femoral artery access after CAG using vascular closure devices (VCDs) is increasingly utilized. In the United States, VCDs are used in an estimated 30% of coronary interventional procedures [3]. Comparisons of closure by VCDs versus MC have shown conflicting results regarding safety and efficacy. Some VCDs have been associated with less oozing, faster ambulation, and better comfort, but no differences in access-site-related major adverse vascular events (MAVE) have been established in a randomized trial or in a systematic meta-analysis [1, 4–6]. VCDs are categorized by their mechanism of action and fall into two main groups: active approximators and passive approximators. Active approximators are devices that physically close the arteriotomy site with use of either a nitinol clip or a suture. Passive approximators are devices that deploy a plug, sealant, or gel at the arteriotomy site without physically closing the arteriotomy.

Randomized data comparing the MynxGrip VCD to MC are few and therefore we performed a randomized study in patients undergoing CAG using the femoral approach comparing the access site closure with MynxGrip VCD to MC.

## Methods

### Design

The CLOSE-UP III study was an investigator-initiated, prospective, randomized, non-blinded single-center trial conducted in a high-volume tertiary interventional heart center in Western Denmark.

### Patients

Consecutive patients scheduled for elective or subacute diagnostic CAG at the Department of Cardiology, Aarhus University Hospital, from June 2014 to May 2017 were included if eligible and if written informed consent could be obtained. The inclusion criteria were age > 18 years, the ability to provide written informed consent, eligibility for CAG using femoral access with a ≤ 7 Fr sheath, possibly including intracoronary pressure measurements or intracoronary imaging. A patient could not be included if percutaneous coronary intervention (PCI) was performed. Patients with a life expectancy of less than one year, patients with puncture and closure at the same site with a closure device < 30 days or with manual compression < 5 days previously were also excluded. Other criteria for exclusion were: ST-elevation myocardial infarction, cardiogenic shock, active infection, multiple punctures, presence of groin hematoma before the closure procedure, known pseudoaneurysm at the femoral artery, prior arterial surgery in abdomen or lower extremities, sheath size > 7 Fr, pregnancy, and simultaneous or planned subsequent femoral vein access.

### Inclusion and randomization

Patients admitted for elective or subacute CAG were informed about potential participation in the study by cardiac care nurses in the ward prior to the procedure. Inclusion was offered at the catheterization laboratory and informed written consent was obtained. Randomization was performed at the end of the CAG, when it was concluded that ad hoc PCI should not be done. A web-based computer randomization was used to allocate patients to the treatment groups at a 1:1 ratio with block randomization stratified by gender and diabetes.

### Procedure

Femoral artery access was obtained by direct puncture using Seldinger technique. CAG was performed according to best practice. Local guidelines were followed for administration of antithrombotic medication. The closure procedure was performed by the operator or by a trained nurse. Operators and nurses were required to have performed ≥ 10 closure procedures using the MynxGrip VCD before performing the closure procedure in study patients. Ultrasound-guided access or femoral angiography prior to the closure procedure was not routinely performed. Use of antithrombotic or anticoagulation therapy was registered. Heparin was used during the procedure at the operator's discretion.

### Closure by MynxGrip

**Fig. 1:**
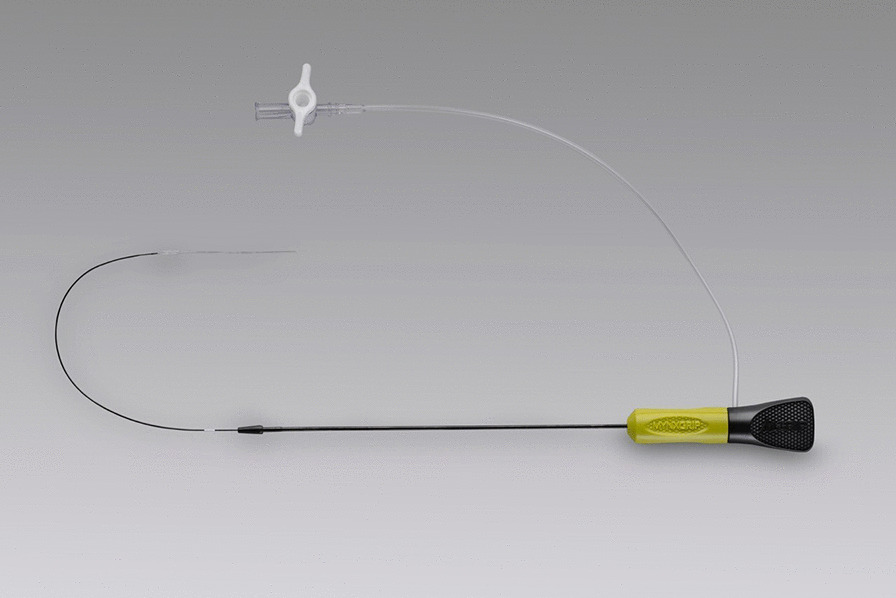
MynxGrip vascular closure device, a passive approximator

**Fig. 2:**
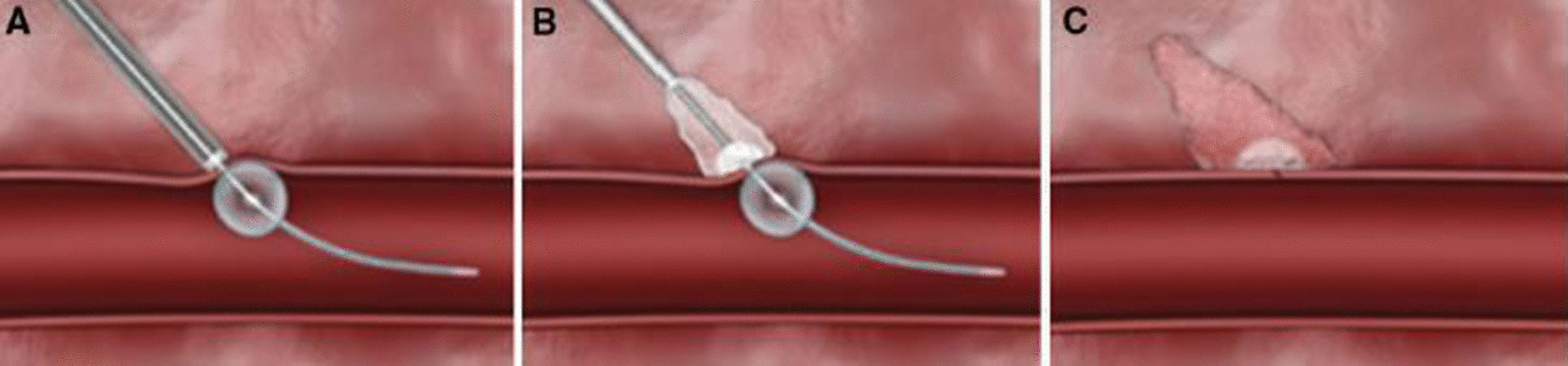
Stepwise illustration of closure of the arteriotomy site with the MynxGrip vascular closure device. A semi-compliant balloon is inflated intraluminally to serve as an anchor (**A**) as the sealant is deployed over the arteriotomy (**B**). The balloon is deflated and removed, leaving the sealant over the arteriotomy (**C**)

MynxGrip (Cardinal Health, Dublin, Ohio) (Fig. 1) is a passive approximator that deploys a polyethylene glycol sealant (hydrogel) over the arteriotomy site. A semi-compliant balloon inflated within the artery serves as an anchor to ensure proper placement. After the sealant is deployed, the balloon is collapsed and removed [1] (Fig. 2). The MynxGrip has been approved to close 5F to 7F arteriotomy sites [7–9].

### Closure by manual compression

Closure by MC is a routine procedure at the study site. The sheath was removed immediately, and MC was applied approximately 1.5 cm proximal to the puncture site by the operator or a nurse trained in MC. Compression was continued for at least five minutes or until hemostasis. The subsequent use of sandbag compression was discouraged to avoid covering the access site, thereby risking unidentified bleeding and hematoma development, and to improve patient comfort.

### Bed rest

As in the CLOSE-UP study [10], one-hour bed rest was recommended for both treatments. During bed rest, the patient was allowed to raise his head to 45 degrees. Ward nurses were instructed to mobilize the patient after one hour of bed rest if no additional bed rest was needed for clinical reasons.

### Complications

Any complication was treated according to local practice. Mild oozing of blood after the closure procedure was treated with an adhesive bandage. A larger bleeding or evolving hematoma were treated by manual compression. In case of persistent or new onset pain after returning to the ward, the patient was examined by a medical doctor. If pseudoaneurysm formation, arteriovenous fistula, femoral stenosis, or retained closure material was suspected, an ultrasound examination was performed to confirm the diagnosis and to guide necessary therapy. Major bleeding was assessed clinically and necessary blood samples including hemoglobin values were obtained. Blood loss was treated according to local practice. In case of clinical signs of blood loss with minor or no demonstrable bleeding the patient was evaluated by a vascular surgeon and a computerized tomography scan performed to detect possible retroperitoneal hematoma.

### Endpoints

The primary endpoint was the 30-day incidence of the combined endpoint MAVE. This includes major access-site related bleeding (Bleeding Academic Research Consortium 3 or 5), ultrasound verified pseudoaneurysm or arteriovenous fistula with an indication for treatment, surgery of ipsilateral leg related to the closure procedure, and infection needing antibiotics.

Secondary endpoints comprised the individual components of the primary endpoint. Others included time to hemostasis defined as cessation of bleeding; incidence of device failure defined as any technical failure of the device or any unsuccessful deployment of the VCD necessitating immediate MC; the rate of vasovagal response defined as sudden onset of reversible nausea, pallor, vomiting and/or loss of consciousness and evidence of bradycardia and/or significant drop in blood pressure during or within five minutes after sheath removal; need for further MC defined as the need rescue MC after initial hemostasis; time to mobilization measured in minutes from the initiation of the closure procedure until the patient was mobilized; the incidence of large hematomas before discharge defined as a palpable groin swelling measuring more than 5 cm at the longest diameter by use of a ruler; and the incidence of Bleeding Academic Research Consortium defined bleedings 1 or 2 within 30 days [11].

### 30-day follow-up questionnaire

Patients were asked to report any contact to the healthcare system and contact to the healthcare system because of major bleeding within 30 days after discharge. If the questionnaire was not returned, the patient was contacted by telephone.

### Statistical analysis

The trial was powered to assess non-inferiority of the MynxGrip compared to MC with respect to the primary endpoint at 30 days. An event rate of 2% was assumed in the MC group based on previous results [12]. With a sample size of 840 patients in each treatment arm, a two-group large-sample normal approximation test of proportions with a one-sided 5% significance level would have 90% power to detect non-inferiority with a predetermined non-inferiority margin of 2%. To account for potential loss to follow-up, we planned to include 2000 patients in the study.

We analysed the data according to the intention-to-treat principle. Continuous variables are presented as mean ± standard deviation, or median values and interquartile range if the distribution did not follow an approximate normal distribution. Categorical variables are expressed as count and percentage. Differences between variables were analysed using Student’s t-test or the Mann–Whitney U test. Categorical variables were analysed using the chi-square test or Fisher’s exact test if any cell numbers are less than 5. A two-sided p-value of less than 0.05 was considered significant. Since only very few numbers of MAVE were found, the regular non-inferiority test was not performed as planned. Instead, the two groups were compared using Fisher’s exact test.

## Results

**Fig. 3:**
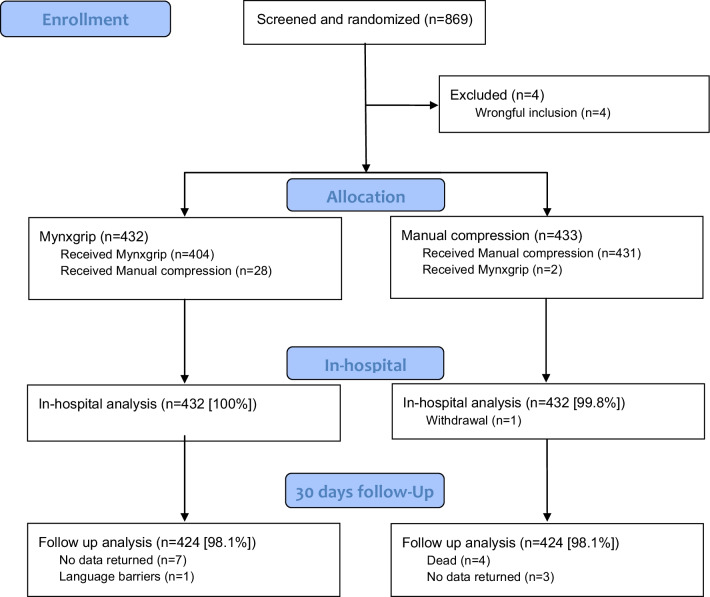
Flow chart of the randomized CLOSE-UP III study and description of patients excluded from analysis

From June 2014 to May 2017, 869 patients were included in the study. The study was stopped prematurely because of low inclusion rate since most operators at the study site changed from femoral to radial approach. Four patients were excluded due to the exclusion criteria and one patient withdrew consent as outlined in the flow chart (Fig. 3) leaving 864 patients with complete in-hospital follow-up. A total of 432 patients underwent closure by MynxGrip VCD and 433 patients by MC. Thirty-day follow-up was available in 424 (98.1%) patients in both patient groups. Four patients in the MC group died within the 30-day follow-up period. Baseline characteristics (Table 1) were balanced in the two groups.

**Table 1:** Baseline characteristics by treatment group

	MynxGrip (n = 432)	Manual compression (n = 432)	*P* value
Age (years)	66 ± 11	66 ± 11	0.99
Gender (male)	285 (66%)	285(66%)	0.99
BMI (kg/m^2^)	26.5 ± 4.2	27.1 ± 4.4	0.07
Diabetes mellitus	67 (18%)	80 (22%)	0.25
Statin treatment	237 (58%)	260 (63%)	0.15
Hypertension	222 (55%)	245 (59%)	0.19
Active smoking	82 (21%)	96 (24%)	0.67
Previous AMI	75 (18%)	70 (17%)	0.64
Previous PCI	92 (22%)	95 (23%)	0.83
Previous CABG	41 (10%)	49 (12%)	0.55
Creatinine level, mmol/L	81 [69:94]	81 [70:95]	0.65
*Antithrombotic therapy*			
Aspirin	85 (19.7%)	82 (19%)	0.80
Clopidogrel	22 (5.1%)	21 (4.9%)	0.88
Ticagrelor	35 (8.1%)	51 (11.8%)	0.07
Fondaparinux	17 (3.9%)	20 (4.6%)	0.61
Dalteparin	10 (2.3%)	6 (1.4%)	0.31
Warfarin	2 (0.5%)	3 (0.7%)	0.99
Heparin	133 (30.8%)	127 (29.4%)	0.66

Values are Mean ± SD, median [interquartile range] or n (%)

No significant differences in the procedural data were found (Table 2).

**Table 2:** Procedural characteristics by treatment group

	MynxGrip (n = 432)	Manual compression (n = 432)	*P* value
Procedure time excl. closure, min	10 [6:17]	10 [6:16]	0.46
Catheter size (5F or 6F)	427 (99.5%)	423 (99%)	0.45
Diastolic blood pressure, mmHg	79 ± 12	77 ± 13	0.18
Systolic blood pressure, mmHg	139 ± 21	136 ± 20	0.05
Local anaesthetic, mg (Lidocain/Bupivacain)	418 (96.8%)	415 (96.1%)	0.58
Benzodiazepin (Midazolam/Nitrazepam)	84 (19.4%)	70 (16.2%)	0.21
Morphine (fentanyl)	18 (4.2%)	18 (4.2%)	0.99
Atropine	8 (1.9%)	6 (1.4%)	0.59

Values are Mean ± SD, median [interquartile range] or n (%)

The primary and secondary endpoints are presented in Table 3. The incidence of the primary endpoint, MAVE after 30 days, was 1.2% (n = 5) in the MynxGrip and 0% (n = 0) in the MC group (p = 0.06). The median time to hemostasis was 4 [3–5] minutes and 10 [7–11] minutes in the MynxGrip group and MC group, respectively (p < 0.0001). The corresponding median times to mobilization was 73 [65–87] minutes and 76 [70–88] (p = 0.01). Device failure was noted in 7% of patients in the MynxGrip group leading to MC in these patients. No further differences in the secondary endpoints were observed. Of the 864 patients included in the study, 140 (16.2%) had subacute CAG because of unstable angina pectoris or non-ST-segment elevation myocardial infarction. There were no differences in the incidence of MAVE when comparing elective patients versus subacute patients; MynxGrip versus MC in stable patients; or MynxGrip versus MC in subacute patients.

**Table 3:** Primary and secondary outcome measures by treatment group

	MynxGrip (n = 432)	Manual compression (n = 433)	*P* value
*MAVE*	5 (1.2%)	0 (0%)	0.06
BARC 3 and 5 bleedings at 30 days	0	0	
Pseudoaneurysm with indication for treatment	2 (0.2%)	0	
Arteriovenous fistula	0	0	
Groin surgery and/or possible related vascular surgery	1	0	
Infection needing antibiotics	2	0	
Time to haemostasis (minutes)	4 [3:5]	10 [7:11]	< 0.0001
Device failure	31 (7%)	–	–
Vasovagal response	1 (0.2%)	2 (0.5%)	0.99
Need for new onset of manual compression	51 (12%)	40 (9%)	0.22
Time to mobilization (minutes)	73 [65:87]	76 [70:88]	0.01
In-hospital large groin haematoma (larger than 5 × 5 cm)	25 (6%)	30 (7%)	0.51
Access site related bleedings	6 (1.4%)	1 (0.2%)	0.12
BARC 1 and 2 bleedings at 30 days	2 [2:2]	1 [1:1]	

Values are Mean ± SD, median [interquartile range] or n (%)

*BARC* Bleeding Academic Research Consortium, *MAVE* major adverse vascular events

## Discussion

Our main findings are that MAVE was rare after closure of femoral arterial access by both the MynxGrip VCD and MC. We found a numerical difference in favour of MC but this did not reach statistical significance. In fact, MAVE was only observed in the MynxGrip group. The MynxGrip device clearly shortened the time to achieve hemostasis.

In general, MAVE was infrequent and no significant differences between the MynxGrip and MC groups were found. These findings are in line with previous studies of VCDs. Three separate meta-analyses reflecting trials related to the early generation of VCD, not including the MynxGrip, showed that VCDs generally performed as well or better than MC controls[4–6]. There was some evidence for an increased risk of groin infection, arterial complications resulting in arterial stenosis, and lower limb ischemia, as well as the need for vascular surgery for repair of arterial complications after the use of VCDs [5]. There were no robust data supporting this conclusion since most studies were underpowered to demonstrate a difference in these rare events. More recent studies comparing MynxGrip to the early generation VCDs have found the devices to be equally safe and efficacious [12, 13,14]. When comparing the MynxGrip to MC and other VCDs, Scott et al. [13, 14] found an increased risk of post-procedural bleeding in the MynxGrip group. However, after adjustment for age, renal failure and sheath size, the difference was no longer significant. Furthermore, at recent large database study of MynxGrip use in PCI compared to other VCDs showed that MynxGrip had marginally higher rate of both vascular complications (1.2% vs. 0.8%) as well as access site bleeding (0.4% vs. 0.3%) and transfusion requirement (1.8% vs. 1.5%). Finally, Resnic et al. [15] analyzed registry data from 73,124 patients who had received MynxGrip after PCI procedures with femoral access and compared outcomes with a propensity-matched concurrent control population who received another VCD. The MynxGrip was associated with a significantly greater risk of any vascular complication than were alternative VCDs; there was also a significantly greater risk of access-site bleeding and transfusion. Whether or not our findings support these findings is unclear. A prospectively designed study with a head-to-head comparison of MynxGrip to other VCDs may be warranted.

The very low incidence of MAVE in the present study may in part reflect a low risk population and the fact that only patients having a diagnostic angiogram without evidence of haematoma before the closure procedure could be randomized. All this said, MAVEs were only seen in the MynxGrip group. Thus, the lack of a statistically significant difference might be caused by the lack of sufficient power in the present study and a difference might be present.

Failure to deploy VCDs is associated with an increased risk of both major and minor vascular complications [16]. The rate of device failure was 7% in this CLOSE-UP III trial. This finding is in line with previous studies showing device failure rates between 5 and 9% [9,12,13,14]. In the present study none of the patients with deployment failure had a MAVE. However, deployment failure was still associated with a high risk of in-hospital large groin haematoma as 6 out of the 31 patients (19.4%) with deployment failure had this complication compared to 6.4% overall.

The CLOSE-UP III trial was conducted before ultrasound guided puncture of the common femoral artery was implemented at our institution. Studies have shown that ultrasound guided cannulation yields both higher success rate at first attempt, fewer inadvertent venous punctures, a shorter access time and lower complication rates [16, 17]. Thus, in an era with increasing utilisation of ultrasound-guided puncture, the results might be different.

## Limitations

Most importantly, the study was stopped prematurely because of a low inclusion rate. Thus, the study does not have the planned power to show non-inferiority of the MynxGrip VCD when compared to MC. A limitation applicable to this and other VCD studies is the lack of blinding. Un-blinded in-hospital endpoint assessment was performed by multiple nurses and doctors, making the assessment less prone to personal bias. It is well known that there is a learning curve using VCDs. MynxGrip was introduced in our catheterisation laboratory shortly before the start of the study. Although all operators were trained and certified in the use of the MynxGrip before they were allowed to include patients in the study, this might have led to underestimation of the true safety and efficacy of MynxGrip.

Like most other randomized trials, we have excluded patients at high risk of femoral artery complications. Although these exclusion criteria are justified by strict study protocol aiming to demonstrate the safety of such devices, cardiologists frequently deal with patients at high risk of bleeding complications because of peripheral vascular disease, obesity, renal insufficiency, hypertension and congestive heart failure. Excluding these patients from the trial may severely prevent conclusive results on this issue. The study was stopped prematurely because of low inclusion rate since most operators at the study site changed from femoral to radial approach. A recent meta-analysis suggested that using the radial approach instead of the femoral approach is associated with a significantly improved outcome [18]. However, radial access is not always possible and femoral access remains commonly used. Our conclusions are not necessarily applicable to patients having PCI since these patients were not included.

## Conclusions

MAVE was rare after closure of femoral arterial access by both the MynxGrip VCD and MC. We found a numerical difference in favour of MC but this did not reach statistical significance. Time to hemostasis was shorter in the MynxGrip group when compared to the MC group.

## Data Availability

The datasets used and/or analysed during the current study are available from the corresponding author on reasonable request.

## Abbreviations

CAG: Coronary angiography
MAVE: Major adverse vascular events
MC: Manual compression
PCI: Percutaneous coronary intervention
VCD: Vascular closure device
